# Acetylcholine from tuft cells promotes M2 macrophages polarization in Hirschsprung-associated enterocolitis

**DOI:** 10.3389/fimmu.2025.1559966

**Published:** 2025-05-09

**Authors:** Ziyi Zheng, Lin Lin, Huifang Lin, Jie Zhou, Zhe Wang, Yang Wang, Jianxin Chen, Caimin Lai, Renfu Li, Zhiyong Shen, Ming Zhong, Cheng Xie, Yinjian Chen, Xuechao Zhang, Zhongjie Guo, Rui Dong, Shiwei He, Feng Chen

**Affiliations:** ^1^ Department of Pediatric Surgery, Fujian Medical University Union Hospital, Fuzhou, China; ^2^ Institute of Population Medicine, School of Public Health, Fujian Medical University, University Town, Fuzhou, China; ^3^ Fuzhou Children’s Hospital Affiliated to Fujian Medical University, Fuzhou, China; ^4^ Fujian Children’s Hospital Affiliated to Fujian Medical University, Fuzhou, China; ^5^ Department of Pediatric Surgery, Children’s Hospital of Fudan University, Shanghai Key Laboratory of Birth Defect, Shanghai, China; ^6^ Key Laboratory of Neonatal Disease, Ministry of Health, Shanghai, China

**Keywords:** Hirschsprung-associated enterocolitis, acetylcholine, tuft cells, macrophages, Hirschsprung’s disease, *Ednrb-/-* mice, *Ednrb-/-*

## Abstract

**Background:**

Hirschsprung-associated enterocolitis (HAEC) is one of the most severe complications in patients with Hirschsprung’s disease (HSCR). Previous research has indicated that acetylcholine (ACH) plays an anti-inflammatory role during inflammation by acting on the α7 nicotinic acetylcholine receptor(α7nAchR) to promote the secretion of anti-inflammatory factors. However, the specific role of ACH in HAEC remains unclear. This experiment aims to explore the sources of ACH in HSCR and its anti-inflammatory mechanisms, thereby identifying new directions for the prevention and treatment of HAEC.

**Methods:**

We analyzed single-cell transcriptome data from HSCR to identify cells that secrete ACH and observed their distribution using immunofluorescence. In *Ednrb-/-* mice, F4/80, iNOS, ARG-1 and CD206 were used to identify and locate M1 and M2 macrophages in different intestinal segments. Western blot, reverse transcription-quantitative polymerase chain reaction, and enzyme-linked immunosorbent assay were used to test the levels of IκBα, tumor necrosis factor-α, interleukin-10, and the macrophage activation pathway proteins JAK2 and STAT3 in different intestinal segments of *Ednrb-/-* mice. Organoid and cell culture techniques were used to verify the anti-inflammatory mechanism of ACH *in vitro* models.

**Results:**

scRNA-seq analysis revealed that tuft cells expressed the CHAT protein. In HSCR, aganglionic segments exhibited heightened cholinergic activity compared with dilated ganglionic segments. In HAEC, inflammation was mainly concentrated in the dilated ganglionic segment and was associated with an increase in M1 macrophages, whereas the aganglionic segment showed less inflammation and was associated with an increase in M2 macrophages. Furthermore, *in vitro* experiments showed that intestinal organoids containing tuft cells promoted an increase in M2 macrophage markers, and ACH promoted M2 macrophage polarization.

**Conclusions:**

Differences in inflammation among various intestinal segments in HAEC may be linked to ACH secreted by tuft cells. Drugs targeting tuft cells have the potential to become important components of HAEC treatment in the future.

## 1 Introduction

Hirschsprung disease (HSCR) is a rare congenital intestinal condition characterized by the absence of ganglion cells in the distal bowel (1, 2). Abnormalities in the proliferation or apoptosis of colonic neural crest cells can contribute to the development of HSCR (3). The most common and serious complication of HSCR is Hirschsprung-associated enterocolitis (HAEC), which is the major cause of mortality (4). Previous studies have identified multiple factors contributing to the progression of HAEC, including alterations in the intestinal flora, abnormal immunity, and a breached intestinal barrier (5–7). However, these factors only partially elucidate the pathophysiological processes in HAEC.

Interactions between epithelial cells are essential for the function and immune defense of mucosal tissues (8, 9), and many substances, including acetylcholine (ACH), serve as bridges between them (10). Although ACH is secreted by nerve cells (11), non-neuronal sources of ACH have also been reported (12, 13). Among the various types of epithelial cells, tuft cells are the primary source of ACH and play a key role in detecting harmful substances in the mucosal layer (14–16). In the respiratory epithelium, tuft cells induce neuronal inflammation, ion transport in neighboring epithelial cells, and ciliary beat frequency via ACH secretion (17, 18). In the intestinal epithelia, ACH released by tuft cells can act on Paneth cells through paracellular pathways, triggering the release of antimicrobial peptides (19).

Although the interactions between tuft and epithelial cells play a significant role in resisting inflammatory invasion (20), immune cells in the lamina propria cannot be overlooked. Previous studies have shown that IL-25 secreted by tuft cells can act on type 2 innate lymphoid cells (ILC2) in the lamina propria to resist helminth infections (21). However, the effects of ACH secreted by tuft cells on other immune cells have not yet been elucidated.

In this study, we focus on the elevation of ACHE in HSCR. By analyzing scRNA-seq data from HSCR and utilizing immunofluorescence (IF) staining, we explore the reasons for the differences in ACH levels in the intestinal epithelium of HSCR patients. Furthermore, we delve into the anti-inflammatory effects of ACH, aiming to provide new methods for the treatment and prevention of HAEC.

## 2 Materials and methods

### 2.1 Patients and samples

Between August 2023 and December 2024, we collected eight colon samples with a pathological diagnosis of HSCR from Fujian Medical University Union Hospital (4 short-HSCR, 2 common-HSCR, 2 long-HSCR). HSCR was diagnosed using preoperative assessments, including traditional anorectal manometry and barium enema evaluation, and the diagnosis was confirmed using postoperative pathological analysis. This study was approved by the Ethics Committee of Fujian Medical University Union Hospital (Approval Number: 2023KJCX006) and was conducted following the government policies and the principles outlined in the Declaration of Helsinki. The parents of all patients signed informed consent forms. Fresh samples were frozen after surgical processing and stored at −80°C.

### 2.2 *Ednrb-/-* mice

Mice model of HSCR. A breeding colony of *Ednrb* ^tm1Ywa/J^ heterozygote mice (*Ednrb* ^tm1Ywa/J^ on a hybrid C57BL/6J–129 Sv background) donated by the Animal Center of Tongji Hospital, Tongji Medical College, Huazhong University of Science and Technology. The animal experimental protocol was approved by the Animal Ethics Committee of the Fujian Medical University Experimental Animal Center (Approval Number: IACUC FJMU 2022-0890). The mice were housed in an SPF-grade environment and had free access to food and water. *Ednrb+/-* mice were obtained through genetic identification after mating with *Ednrb+/+* mice. Once sexually mature, *Ednrb+/-* female and male mice were crossed to generate *Ednrb-/-* mice. When *Ednrb-/-* mice exhibited signs of abdominal distension, inability to expel feces, lethargy, and significant weight loss, they were euthanized via intraperitoneal injection of an overdose of 1% pentobarbital (0.3 mL/20 g). Their intestines were dissected for further analysis. This experiment involved 18 *Ednrb-/-* mice. Of these, eight developed HAEC (3 weeks old) and were included in the experimental group for further study, Littermate 3-week-old *Ednrb+/+* mice were used as the control group.

### 2.3 Benzalkonium-treated rat model

The animal experimental protocol was approved by the Animal Ethics Committee of the Fujian Medical University Experimental Animal Center (Approval Number: IACUC FJMU 2024-0029). Sixteen adult female Sprague–Dawley rats were randomly assigned to two groups: an intervention group and a saline control group with eight rats each. The rats were fasted for 1 day prior to surgery to minimize intestinal contents and reduce the risk of aspiration during anesthesia. On the following day, they were anesthetized with 2% sodium pentobarbital administered via intraperitoneal injection (1.0 mL/400 g). Once the rats lost their muscular reflexes and were fully anesthetized, the abdominal cavity was opened. In the BZK group, filter paper soaked in 0.1% BZK solution was wrapped around the colon, whereas in the control group, filter paper soaked in saline was used. The filter paper was replaced every 5 min for a total of six changes. After the final change, the abdominal cavity was irrigated with saline solution and sterilized. Post-surgery, the rats received intraperitoneal injections of gentamicin sulfate (100 mg/kg body weight) prophylactically to prevent infections. After 2 weeks, the mice were euthanized using 2% pentobarbital sodium (0.4 mL/100 g); dilated and narrow intestinal segments were collected. The location of the ganglia was observed using immunohistochemical (IHC) staining for calretinin. In the BZK-treated group, segments with ganglia were classified as dilated ganglionic segments, whereas those without ganglia were classified as BZK-treated segments. The control group was designated the healthy control.

### 2.4 Quality control and batch effects correction of scRNA-seq data

We analyzed HSCR single-cell data (22), which included eight aganglionic and five ganglionic intestinal segments from seven patients with HSCR (2 short-HSCR, 1 common-HSCR, 1 long-HSCR, and 3 total colonic aganglionosis), as well as 29 healthy segments from seven healthy donors. In total, we analyzed 92,958 cells, including 59,594 ganglionic segments and 33,364 normal segments.

Gene-barcode matrices were converted into a Seurat object using the “Seurat” R package (version 4.0.2) (23). Each HSCR tissue sample was filtered to remove cells with fewer than three genes expressed, as well as cells that expressed fewer than 500 genes. Cells with mitochondrial and hemoglobin genes were removed to filter out low-quality and potential red blood cells. Subsequently, 13 HSCR tissue sample count matrices were merged. To account for differences in sequencing depth across samples, the SCTransform R package (version 0.3.2) was used to normalize the expression values for the total unique molecular identifier counts per cell. PLCG2 (24), mitochondrial, and ribosomal genes were removed from the highly variable genes, as they contributed to the highest variability in the merged dataset, consistent with a previous study (25).

### 2.5 Dimensionality reduction and clustering

Clustering was performed and visualized using the uniform manifold approximation and projection (UMAP) algorithm for harmony dimensionality reduction via the Seurat function, RunUMAP. Marker genes described in previous studies were used to categorize the cells into known biological cell types (25, 26). A series of visualization maps were used to locate the expression sites of CHAT.

### 2.6 Western blot analysis

Colonic tissues were obtained from *Ednrb-/-* and *Ednrb+/+* mice and ground in RIPA buffer (Epizyme, China) containing 1% protease inhibitor (Epizyme, China) and 2% phosphatase inhibitors (Epizyme, China). The protein concentration was determined using a BCA protein assay kit (Epizyme, China), and the samples were prepared for electrophoresis. The extracted total protein was separated using SDS-PAGE and transferred onto a PVDF membrane. The PVDF membrane was blocked with 5% skim milk at room temperature. After blocking, the membrane was incubated overnight at 4°C with a mixture of diluted primary antibodies, including iNOS (Proteintech, 1:1000), TNF-α (Proteintech, 1:2000), IκBα (Proteintech, 1:5000), p-IκBα (Proteintech, 1:5000), CD206 (Proteintech, 1:2000), CD68 (Proteintech, 1:5000), CHAT (Proteintech, 1:1000), P-STAT1 (CST, 1;2000), P-STAT6 (CST, 1;2000) JAK2 (ABclonal,1;2000), P-JAK2 (Abcam, 1;2000), STAT3 (Abcam, 1;2000), P-STAT3 (Proteintech, 1:1000), GAPDH (Proteintech, 1:50000), and β-actin (Proteintech, 1:50000). The following day, the membrane was incubated with a TBST-diluted secondary antibody (Beyotime, China) for 1 h at room temperature. Post-incubation, it was washed three times with TBST at room temperature for 5 min each time. Protein bands were visualized using Omni-ECL (Epizyme, China), and detected using the ChemiDoc MP multifunctional imaging system (Bio-Rad, USA). Protein band densities were analyzed using the ImageJ version 1.8.0 software (National Institutes of Health).

### 2.7 Reverse transcription-quantitative polymerase chain reaction analysis

Colonic tissues were obtained from patients, *Ednrb-/-* mice, and healthy control mice (the latter groups were sacrificed prior to tissue collection) and mechanically milled for homogenization, and total mRNA was isolated using RNAiso Plus (Takara, Japan). The concentration of the isolated total mRNA was measured at 260 nm using a spectrophotometer (Thermo USA), and its integrity was assessed using Agarose gel electrophoresis. Total cDNA was synthesized from the isolated mRNA according to the instructions provided with the PrimeScript™ RT reagent Kit (Takara, Japan). Subsequently, the TB Green Premix EX Taq kit (Takara, Japan) and specific primers for the genes were added to prepare the reaction mixture for quantitative polymerase chain reaction. The quantitative polymerase chain reaction was performed as follows: An initial denaturation step was carried out at 95°C for 2 min, followed by 40 cycles of reverse transcription-quantitative polymerase chain reaction. Each cycle included DNA denaturation at 95°C for 5 s and primer annealing/extension at 60°C for 34 s. The primer sequences are listed in the attached **Table 1**.

### 2.8 Enzyme-linked immunosorbent assay

ACH and IL-10 levels were measured using a commercially available ELISA kit (Elabscience, China). Following euthanasia, the colonic tissues were quickly weighed on an ice platform and nine times the volume of cold normal saline was added to generate a 10% homogenate. Subsequently, 0.8 mL of the homogenate was collected, and 1.4 mL of pure water was added, followed by 0.2 mL of 1.54 mmol/L physostigmine sulfate. The sample was centrifuged for 10 min at 4 °C and 3500 r/min, and the supernatant was stored in a refrigerator at -20 °C until testing. The ACH content was determined using Hestrin alkaline hydroxylamine colorimetry (27) according to the manufacturer’s instructions, the same thing with macrophage cell line.

### 2.9 Hematoxylin and eosin staining

The obtained intestinal tissues were embedded and sliced. After dewaxing and hydration, hematoxylin and eosin staining was performed using a hematoxylin and eosin staining kit (Solarbio, China) and observed under a light microscope.

### 2.10 IHC staining

The tissue sections were dewaxed and rehydrated using a series of xylene washes and an ethanol gradient to remove any waxy substances and gradually restore the hydration state of the tissues. Each section was fixed in 4% paraformaldehyde (Biosharp, Canada) for 30 min. Antigen retrieval was performed using Tris-EDTA buffer (Solarbio, China). Each slide was treated with a peroxidase-blocking solution (Genentech, China) for 30 min. Membranes were permeabilized using 1% Triton X-100 (Solarbio, China). Subsequently, slides were blocked with 10% goat serum (Solarbio, China) for 1 h, and incubated overnight at 4°C with primary antibodies, including Calreticulin (Proteintech, 1:500) and acetylcholinesterase (ACHE) (Proteintech, 1:500). The following day, the corresponding secondary antibody was applied, followed by DAB (Genetech, China) and hematoxylin staining (Solarbio, China). The slides were observed under a microscope, and density analysis was performed using ImageJ version 1.8.0 (National Institutes of Health).

### 2.11 IF staining

After baking the sections in a 60°C constant temperature oven for 1 h, they were dewaxed and hydrated using a series of xylene and graded ethanol series. Each section was fixed with 4% paraformaldehyde (Biosharp, China) for 30 min and washed with phosphate buffer saline (PBS). Antigen retrieval was performed using Tris-EDTA (Solarbio, China) at 95°C, followed by another PBS wash. Permeabilization was achieved by incubating the sections with 0.1% Triton X-100 (Solarbio, China) for 10 min. Subsequently, the sections were blocked with 10% goat serum (Solarbio, China) for 1 h. The primary antibodies used were CHAT (Proteintech, 1:200), DCLK1 (Proteintech, 1:200), F4/80 (CST, 1:600), iNOS (Proteintech, 1:500), CD206 (Proteintech, 1:500), and α7nAchR (Proteintech, 1:200). These antibodies were incubated with the sections overnight at 4°C. The following day, the sections were washed and stained with fluorescent secondary antibodies (goat anti-rabbit Alexa Fluor 488, goat anti-mouse Alexa Fluor 647, and goat anti-rabbit Alexa Fluor 555; 1:200). After DAPI (Sigma, Germany) staining, the sections were mounted using an anti-fade mounting medium and imaged using a super-resolution confocal microscope (TCS SP8).

### 2.12 Flow cytometry

Mouse colonic tissue was rinsed in PBS, cut into small pieces, and placed in RPMI 1640 medium containing 0.2 mg/mL collagenase IV and 0.05 mg/mL DNAase I. The tissue fragments and digestive enzymes were transferred into a centrifuge tube and incubated in a shaking water bath at 37°C for 40 min. Digestion was stopped by supplementing the medium with 5% FBS. After filtering through a 40-μm cell strainer and washing twice with PBS (400 g, 4 min), a mouse colon single-cell suspension was obtained. The cells were blocked with 2% BSA at 4°C for 30 min, washed with PBS, and incubated with antibodies F4/80 (Biolegend APC) and CD206 (Biolegend FITC) in the dark for 45 min. After centrifugation (400g, 4 min), the cells were fixed with 4% paraformaldehyde for 10 min, permeabilized with 0.25% Triton X-100 for 10 min, then incubated with antibody iNOS (Biolegend PE) in the dark for 45 min. Finally, the cells were washed and resuspended in 200 μL PBS for flow cytometry analysis.

### 2.13 Organoid culture

C57BL/6 mice aged 6–8 weeks were euthanized with an intraperitoneal injection of 1% pentobarbital sodium (0.5 mL/20 g). Once respiratory and cardiac arrest were confirmed and muscle reflexes ceased, the upper one-third of the cecum was excised for subsequent organoid experiments. The intestinal tissue was placed in a culture dish on ice, the connective tissue was removed, the lumen was opened longitudinally, and the tissue was washed with cold DPBS (Gibco, USA) to remove the luminal contents. The tissues were cut into pieces and washed with cold DPBS containing 1% double antibiotics. Tissue fragments were incubated with 0.02% EDTA cell dissociation solution (Beyotime, China) on ice for 15 min. After removing EDTA, the tissue fragments were resuspended in pre-cool DPBS and filtered through a 70 μm cell strainer to isolate the crypts. Following centrifugation at 300 g for 5 min, the crypts were resuspended in Matrigel (Corning, USA). The crypts and Matrigel mixture were seeded at 50 μL per well in a 24-well plate, and 500 μL of organoid culture medium was added to each well. The culture medium contained Advanced DMEM/F12 (Gibco, USA), L-Glutamine (1x, Yeasen, China), Hepes (0.01M, Procell, China), Penicillin (10 kU/mL)-streptomycin (10 mg/mL) (1x, Procell, China), N2 supplement (1x, Yeasen, China), B27 supplement (1x, Yeasen, China), n-Acetylcysteine (1.25 mM, Yeasen, China), EGF (0.05 μg/mL, Yeasen, China), R-spondin1 (500 ng/mL, Yeasen, China), Noggin (100 ng/mL, Yeasen, China), and Wnt3a (100 ng/mL, Yeasen, China), IL-13 protein (20ng/ml, MCE, USA) Subculture was performed after the organoids grew to a diameter exceeding 400 μm.

### 2.14 Cell culture

RAW264.7 cells (Procella China) were cultured in DMEM medium (Procella China) containing 10% Fetal Bovine Serum (Vivacell China) and 1% Penicillin(10 kU/mL)-streptomycin(10 mg/mL) (Procella China). The cells were maintained at 37°C in a 5% CO₂ incubator. Once cells reached 80% confluence, they were routinely passaged. After three passages, the cell morphology was observed under a microscope to ensure stability before proceeding with the experiments. RAW264.7 cells were pretreated with LPS (Sigma, 0.5 μg/mL) for 2 h, followed by stimulation with α7nAchR stimulant (MCE, 10 μM), ACH (Sigma, 1 μM), and α7nAchR inhibitor (Tocris, 10 μM) for 22 h.

### 2.15 Immunocytochemistry

RAW264.7 cells (0.05×10^6^) were seeded in polylysine-coated culture dishes, followed by drug treatment. The cells were treated with 4% paraformaldehyde for 10 min, permeabilized with 0.1% Triton X-100 for 10 min, and blocked with 10% goat serum for 1.5 h. Primary antibodies, including CD206 (Proteintech,1:200) and iNOS (Proteintech,1:200), were incubated with the cells overnight at 4°C. The following day, fluorescent secondary antibodies (Abcam, goat anti-rabbit Alexa Fluor 488, goat anti-mouse Alexa Fluor 647, 1:200) were added, followed by DAPI (Sigma, USA) staining. Images were captured using a confocal laser scanning microscope.

### 2.16 Statistical analysis

Data analysis and plotting were performed using GraphPad Prism 8.4 software. First, perform a normality test. If the data followed a normal distribution, paired t-tests were used to compare intestinal tissues from *Ednrb-/-* mice with those from healthy control mice, as well as to compare ganglionic dilated segments with aganglionic segments in patients with HSCR. Statistical significance was set at p < 0.05. If not, apply Wilcoxon signed-rank test, Statistical significance was set at p < 0.05. Additionally, cell experiment data were analyzed using Mann-Whitney test, with the significance level set at p < 0.05.

## 3 Results

### 3.1 Cholinergic system in intestinal epithelium of aganglionic segments is active

**Figure 1:**
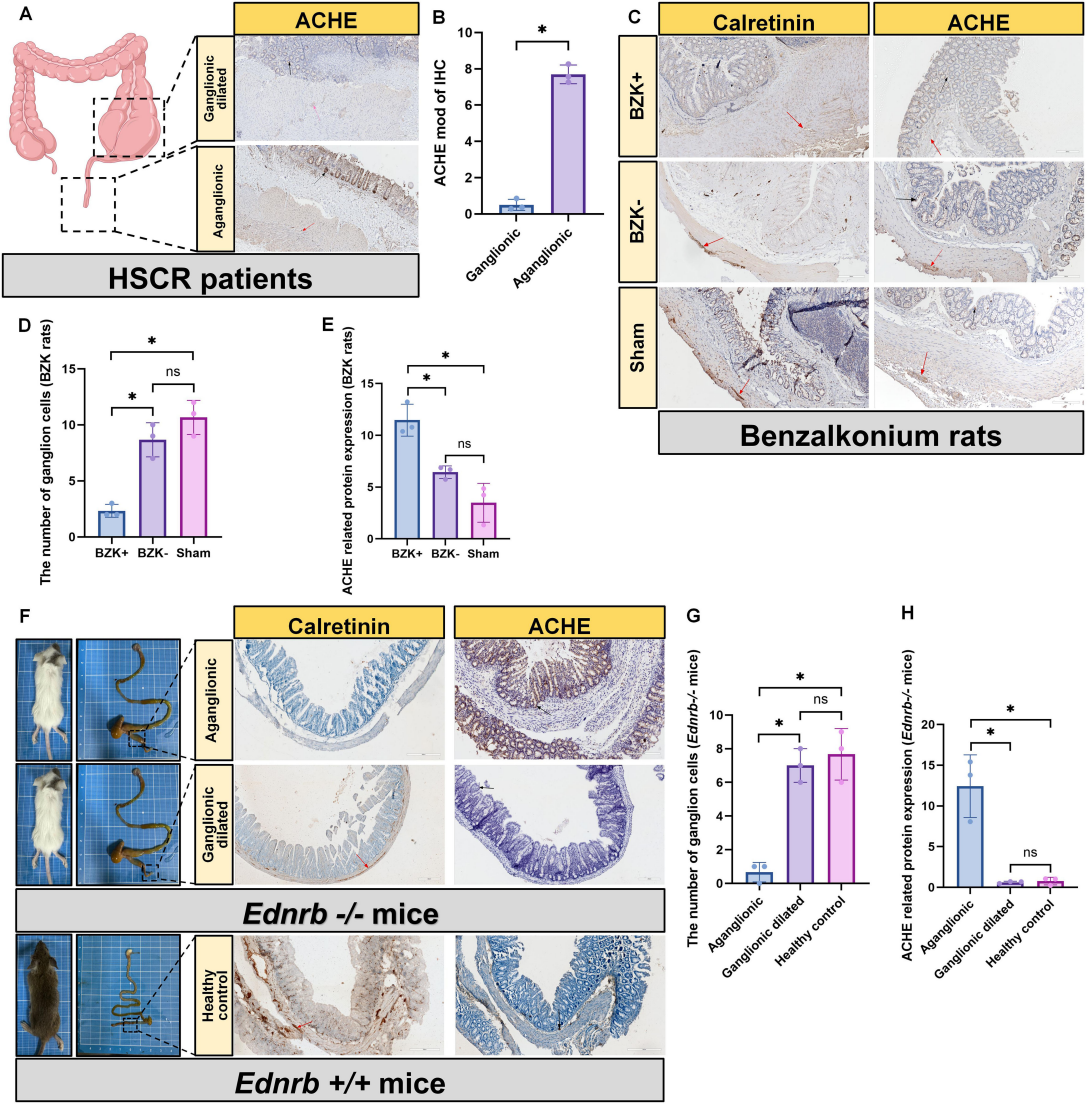
Increased cholinergic activity in the intestinal epithelium of aganglionic segments. **(A)**: IHC staining for the detection of ACHE in ganglion dilated segments and aganglionic segments in HSCR (n=3). Scale bar: 200 μm. Black arrow: the location of ACHE, Red arrow: the location of ganglion cell. **(B)**: Statistical analysis of intestinal tissues from patients with HSCR (n=3) by ACHE IHC. The data conform to a normal distribution. Paired t-test (*: P<0.05). **(C)**: IHC staining for the detection of calretinin and ACHE in BZK untreated segments (BZK-) and BZK treated segments (BZK+) in rats and Sham group (n=3), scale bar: 200 μm. Black arrow: the location of ACHE, Red arrow: the location of ganglion cell. **(D)**: Statistical analysis of ganglion cell counts in BZK rats (n=3) using calretinin IHC. The data conform to a normal distribution. Paired t-test (*: P<0.05, ns: P>0.05). **(E)**: Statistical analysis of intestinal tissues from BZK rats (n=3) by ACHE IHC. The data conform to a normal distribution. Paired t-test (*: P<0.05, ns: P>0.05). **(F)**: IHC staining for the detection of calretinin and ACHE in ganglion dilated segments and aganglionic segments in *Ednrb-/-* mice (n=3). Arrows: ganglia c ells, scale bar: 200 μm. Black arrow: the location of ACHE, Red arrow: the location of ganglion cell. **(G)**: Statistical analysis of ganglion cell counts in *Ednrb-/-* mice (n=3) and *Ednrb+/+* mice (n=3) using calretinin IHC. The data conform to a normal distribu tion. Paired t-test (*: P<0.05, ns: P>0.05). **(H)**: Statistical analysis of intestinal tissues from *Ednrb-/-* mice (n=3) and *Ednrb+/+* mice (n=3) by ACHE IHC. The data conform to a normal distribution. P aired t-test (*: P<0.05, ns: P>0.05).

HSCR was previously diagnosed by observing positive expression of ACHE in aganglionic segments through IHC staining, indicating the presence of numerous abnormally activated cholinergic systems in the aganglionic segments (28–30). To determine the cholinergic distribution, we performed similar experiments related to ACHE. In patients with HSCR, the expression of ACHE was significantly elevated in aganglionic segments compared to that in ganglionic dilated segments, and was abundantly present in the epithelial layer of the aganglionic segments (**Figures 1A, B**). To verify whether the same phenomenon occurs in animal models, we performed ACHE IHC staining. We found that abundant positive ACHE expression in the intestinal epithelium of the aganglionic segments of BZK rats (**Figures 1C–E**) and *Ednrb-/-* mice (**Figures 1F–H**), compared with ganglionic dilated segments and sham controls.

### 3.2 Cholinergic activity in intestinal epithelium of aganglionic segments is associated with tuft cells

**Figure 2:**
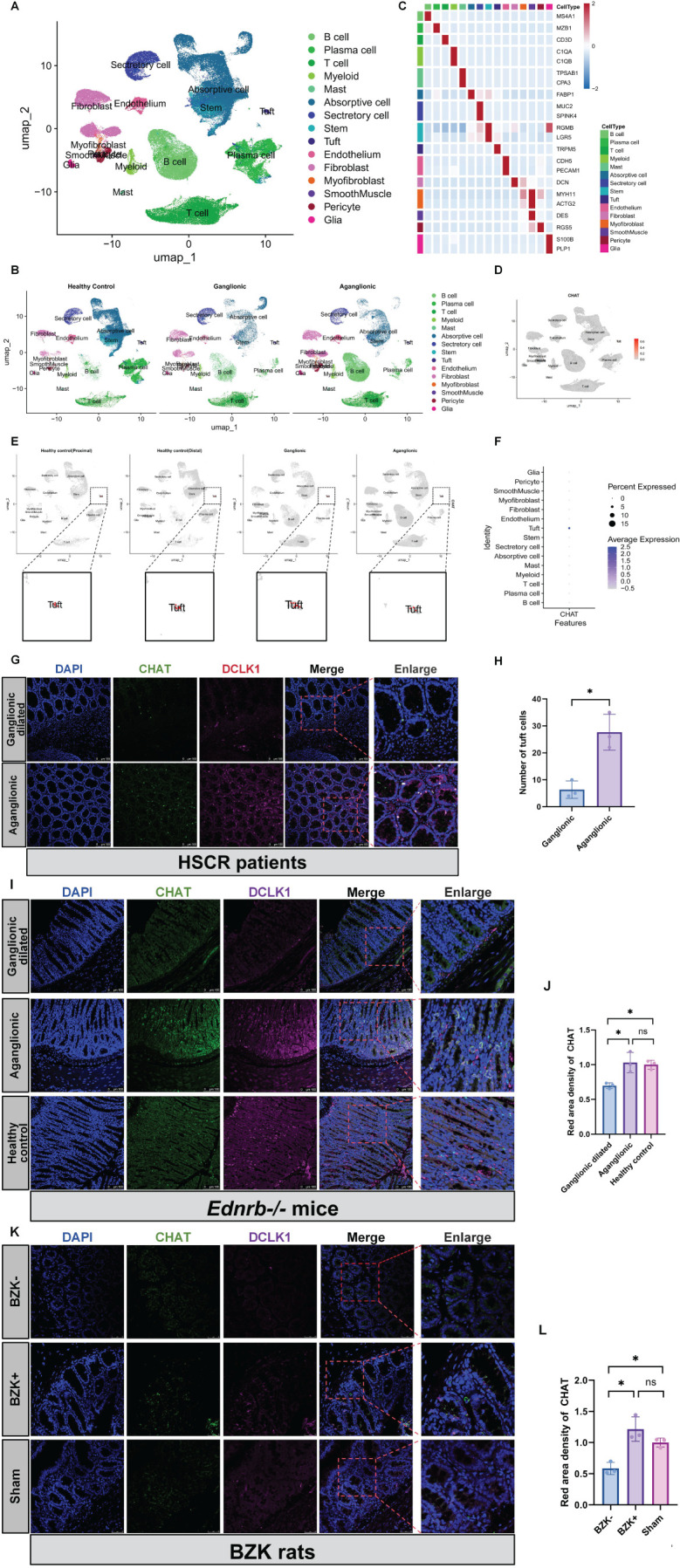
Increased ACH in aganglionic segments is associated with tuft cells. **(A, B)**: Perform UMAP projection of scRNA-seq data comparing patients of HSCR (n=5) and healthy controls (n = 7). Display separately the scRNA -seq samples from healthy controls, ganglionic segments, and aganglionic segments. **(C)**: Create a Heatmap showing the relative expression of marker genes in merged datasets of cells, with color bars corresponding to different cell-type groups. **(D, E)**: The localization of CHAT within cell clusters across different groups. **(F)**: A bubble chart was used to localize the expression levels of CHAT within different cell clusters. **(G)**: Localization of DCLK1 and CHAT proteins in the aganglionic(n=3) and ganglionic-dilated(n=3) segments of the patience of HSCR. Red, DCLK1; Gre en, CHAT; Blue, DAPI. Scale bars: 100 μm. **(H)**: Statistical analysis of number of tuft cells in intestinal tissues of patients with HSCR. The data conform to a normal distribution. Paired t-test (*: P<0.05). **(I)**: Localization of DCLK1 and CHAT proteins in the aganglionic(n=3) and ganglionic dilated(n=3) segments of the *Ednrb-/-* mice and *Ednrb+/+* mice colo n. Red, DCLK1; Green, CHAT; Blue, DAPI. Scale bars: 100 μm. **(J)**: Statistical analysis of CHAT IF optical density in intestinal tissues of *Ednrb-/-* mice and *Ednrb+/+* mice. All fluorescence intensities across the groups have been normalized to DAPI. Optical density values from the aganglionic(n=3) and ganglionic dilated(n=3) segments were normalized against those of the healthy control group. The data conform to a normal distribution. Paired t-test (*: P<0.05). **(K)**: Localization of DCLK1 and CHAT proteins in the BZK+(n=3) and BZK-(n=3) segments of the BZK rats and healthy control. Pink, DCLK1; Green, CH AT; Blue, DAPI. Scale bars: 50 μm. **(L)**: Statistical analysis of CHAT IF optical density in intestinal tissues of BZK rats. All fluorescence intensities across the groups have been normalized to D API.. Optical density values from BZK+ and BZK- groups were normalized against those of the healthy control group. The data conform to a normal distribu tion. Paired t-test (*: P<0.05).

Cholinergic activity is associated with ACH (31). To investigate the ability of intestinal epithelial cells to synthesize ACH, we analyzed single-cell data from five ganglionic segments and eight aganglionic segments derived from seven patients with HSCR (2 short-HSCR, 1 common-HSCR, 1 long-HSCR, and 3 total colonic aganglionosis), as well as 29 healthy intestinal segments from seven healthy donors. After quality control and doublet exclusion, a total of 92,958 cells from patients with HSCR and 64,045 cells from healthy donors were analyzed. Using UMAP for dimensionality reduction, and classic gene markers for cell annotation, we successfully visualized the distribution of cell clusters in HSCR single-cell RNA-seq (**Figures 2A–C**). By localizing CHAT, a key enzyme for ACH synthesis, in the single-cell RNA-seq of patients with HSCR, we found that CHAT was only expressed in tuft cells of the intestinal epithelial cells (**Figures 2D–F**). Moreover, in patients with HSCR, *Ednrb-/-* mice, and BZK-treated rats, tuft cells were predominantly scattered at the basal part of the intestine, particularly in aganglionic segments (**Figures 2G–L**).

### 3.3 Inflammation in HAEC mainly occurs in ganglionic segments

**Figure 3:**
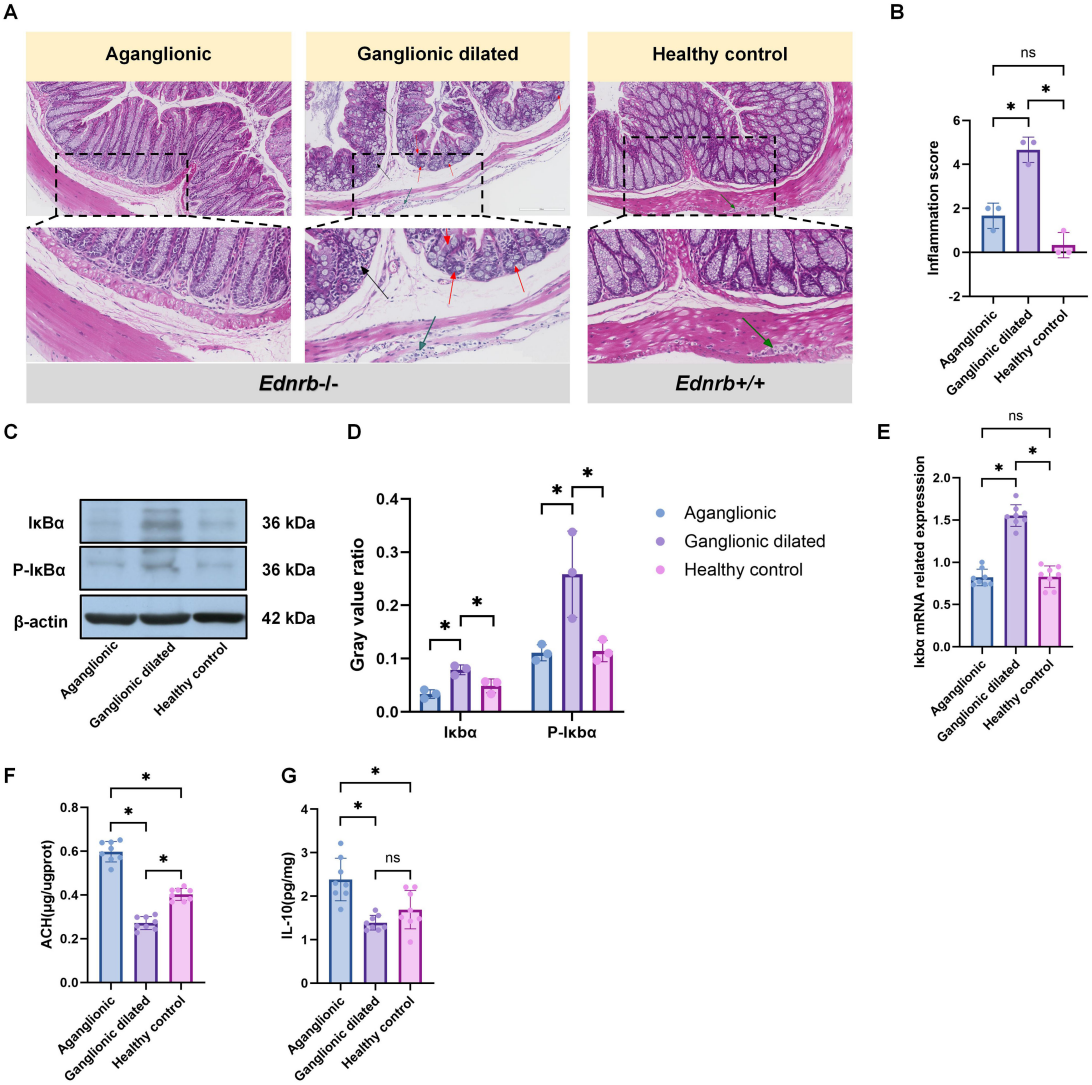
The inflammation in HAEC is mainly concentrated in the dilated ganglion segment. **(A)**: HE staining images corresponding to different intestinal segments (n=3). Red arrows: crypt abscess, black arrows: immune cell infiltration, green arrows: ganglion cell. Scale bars: 200 μm. **(B)**: Statistical analysis of intestinal inflammation scores in HE staining. Data shown represent results from 3 independent experiments. The data do not conform to a normal distribution. Wilcoxon Test (*: P<0.05). **(C)**: Expression of inflammation-related proteins, including Ikba and p-Ikbo, in different intestinal segments(n=3). **(D)** Semiquantitative analysis of protein expression levels, with each protein being normalized to β-actin. The data conform to a normal distribution. Paired t- test (*: P<0.05). **(E)**: Gene expression related to *Iκbα* in different intestinal segments of *Ednrb-/-* mice (n=8). The data do not conform to a normal distribution. Wilcoxon Tes t(*: P<0.05, ns: P>0.05). **(F)**: ELISA experiment to detect the expression levels of IL-10-related proteins in different intestinal segments of *Ednrb-/-* mice (n=8). The data do not conf orm to a normal distribution. Wilcoxon Test (*: P<0.05, ns: P>0.05). **(G)**: ELISA experiment to detect the expression levels of ACH in different intestinal segments of *Ednrb-/-* mice (n=8). The data do not conform to a normal distribution. Wilcoxon Test (*: P<0.05).

To explore the relationship between tuft cells and other immune cells in HAEC, we investigated the sites of inflammation. Because *Ednrb-/-* mice typically develop enterocolitis in the third week, we chose them for subsequent research. In the third week, significant immune cell infiltration and the appearance of crypt abscesses were observed in the ganglionic dilated segment of *Ednrb-/-* mice. However, these features were absent in the aganglionic segments and the healthy control group (**Figure 3A**). Pathological scoring (32) of inflammatory damage to intestinal tissues revealed that the small bowel colitis score was highest in the dilated colon **(** **Figure 3B** **)**, Furthermore, the expression of the inflammation-related gene and protein Iκbα was increased in the ganglionic dilated segment but decreased in the aganglionic segment (**Figures 3C–E**). Conversely, the anti-inflammatory factor, IL-10, showed increased expression in the aganglionic segment and decreased expression in the dilated ganglionic segment (**Figure 3F**). Notably, ACH expression increased in the aganglionic segment (**Figure 3G**). Our findings indicate that inflammation in HAEC was mainly concentrated in ganglionic dilated segments, whereas inflammation in aganglionic segments was relatively mild and correlated with ACH expression.

### 3.4 Inflammation in HAEC is related to the polarization of intestinal macrophages

**Figure 4:**
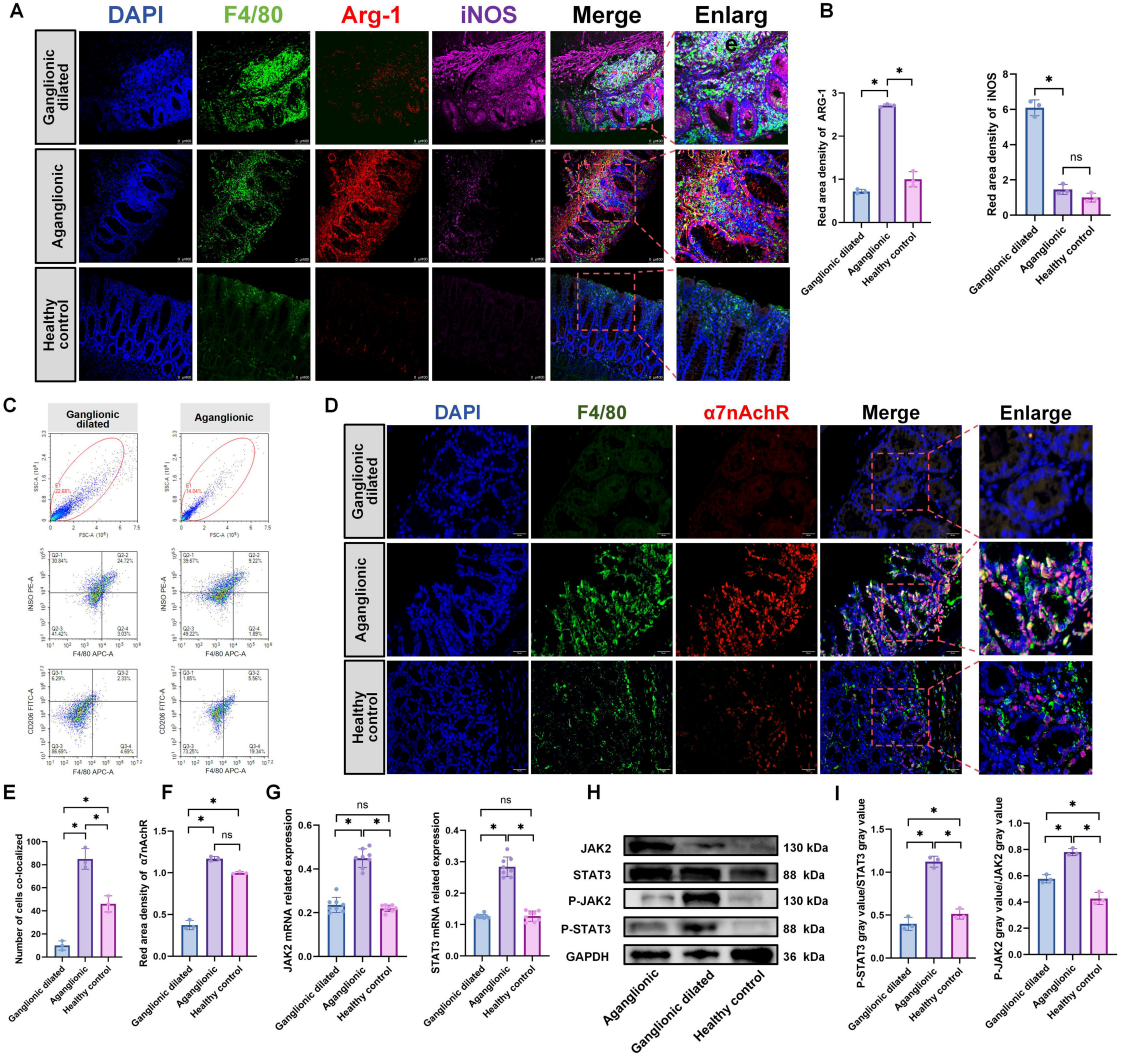
M2 macrophages is elevated in the aganglionic intestinal segment of 3-week-old *Ednrb-/-* mice. **(A)**: IF staining of F4/80, CD206, and iNOS to identify M1 and M2 macrophage in different intestinal segments of *Ednrb-/-* mice (n=3). Green, F4/80; Red, ARG-1; Pink, iNOS; Blue, DAPI. Scale bars: 100 μm. **(B)**: Statistical analysis of ARG-1 and iNOS IF optical density in intestinal tissues of *Ednrb-/-* (n=3). All fluorescence intensities across the groups have been normalized to DAPI. Optical density values from the aganglionic(n=3) and ganglionic dilated(n=3) segments were normalized against those of the healthy control group. The data conform to a normal distribution. Paired t-test (*: P<0.05). **(C)**: Flow cytometry analysis of distinct macrophage subset proportions in the intestines of *Ednrb-/-* mice. APC: F4/80, PE: iNOS, FITC: CD206 **(D)**: IF staining of α7nAChR and F4/80 to colocalizate in macrophages in different intestinal segments (n=3). Red, α7nAChR; Green, F4/80; Blue, DAPI. Scale bars: 50 μm. **(E)**: Count the number of cells co-localizing F4/80 and α7nAchR. The data conform to a normal distribution. Paired t-test (*: P<0.05). **(F)**: Statistical analysis of α7nAchR IF optical density in intestinal tissues of *Ednrb-/-* (n=3). All fluorescence intensities across the groups have been normalized to DAPI. Optical density values from the aganglionic(n=3) and ganglionic dilated(n=3) segments were normalized against those of the healthy control group. The data conform to a normal distribution. Paired t-test (*: P<0.05). **(G)**: The mRNA levels of *JAK2* and *STAT3* by RT-qPCR in different intestinal segments of *Ednrb-/-* mice (n=8). The data do not conform to a normal distribution. Wilcoxon Test (*: P<0.05, ns: P>0.05). **(H, I)**: Western blot analysis of colonic proteins in different intestinal segments(n=8). Semiquantitative analysis of protein expression levels, with each protein being normalized to GAPDH, and compare the grayscale of phosphorylated protein to total protein to observe phosphorylation levels. Data shown represent results from 3 independent experiments. The data do not conform to a normal distribution. Wilcoxon Test (*: P<0.05, ns: P>0.05).

The relationship between HAEC and macrophages was confirmed (33, 34). Our study results revealed that macrophages in the dilated ganglionic segment were predominantly of the M1 type, whereas those in the aganglionic segment were mainly of the M2 type (**Figures 4A, B**), Flow cytometry analysis also revealed increased expression of M2 macrophages (marker: CD206) in aganglionic bowel segments (Q2+Q4 quadrants), while M1 macrophages (marker: iNOS) were elevated in dilated ganglionic segments (Q2+Q4 quadrants)(**Figure 4C**). Given the high expression of ACH in the aganglionic segment, we assessed the expression of α7nAchR in this segment. The result showed that the expression level of α7nAchR was significantly high in the aganglionic segment compared to that in the ganglionic dilated segment, and it co-localized with macrophages (**Figures 4D–F**). Furthermore, the JAK2-STAT3 signaling pathway was activated in the aganglionic segment (**Figures 4G–I**).These findings suggest that macrophages in aganglionic segments receive ACH signals, activating the JAK2-STAT3 signaling pathway and subsequently promoting M2 polarization.

### 3.5 Tuft cells promote the increase of M2 macrophage markers

**Figure 5:**
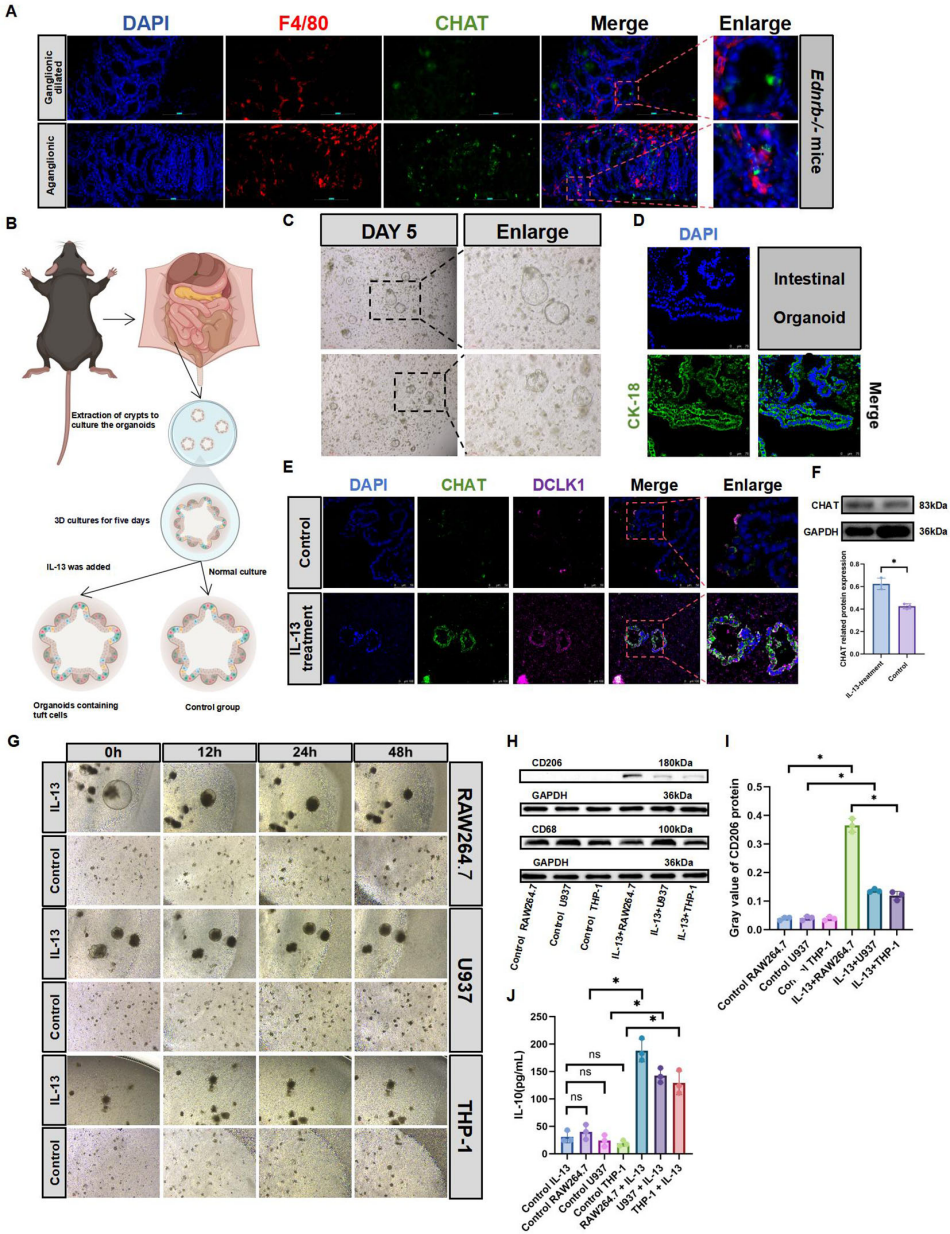
Tuft cells promote macrophage M2 polarization. **(A)**: IF staining of F4/80 and CHAT to identify tuft cell and macrophage (n=3). Red, F4/80; green, CHAT; blue, DAPI.Scale bars: 100 μm. **(B)**: Diagram of the organoid culture model. Addition of IL-13 on the fifth day promoted organoid differentiation into tuft cells **(C)**: Culture the organoids normally, and passaged them when vacuoles were > 400μm for further experiments. Day 5 of organoid culture: diameter >100μm. Scale bars: 100 μm. **(D)**: IF identification of organoids on the fifth day of normal culture. Green, Cytokeratin-18 (CK-18); blue, DAPI. Scale bars: 75 μm. **(E)**: IF identification of tuft cell expression in intestinal organoids treated with IL-13 (20 ng/mL) for 3 days. Red, DCLK1; green, CHAT; blue, DAPI. Scale bars: 100 μm. **(F)**: Expression of CHAT protein in IL-13-treated organoids. Semiquantitative analysis of protein expression levels, with each protein being normalized to GAPDH. The data conform to a normal distribution. Paired t-test (*: P<0.05). **(G)**: On Day 5 of intestinal organoid culture, IL-13 is added. On Day 8, macrophages are co-cultured for 2 days without removing the Matrigel. Observations of macrophage aggregation and the status of intestinal organoids are conducted at Oh, 12h, 24h, and 48h. RAW264.7 uses DMEM culture medium; U937 and THP-1 use 1640culture medium. **(H, I)**: Western blotting analyzed CD206 and CD68 expression in co-cultured macrophages. Semiquantitative analysis of protein expression levels, with each protein being normalized to GAPDH. Data shown represent results from 3 independent experiments. The data do not conform to a normal distribution. Wilcoxon Test (*: P<0.05, ns: P>0.05). **(J)**: ELISA analysis of IL-10 secretion in co-culture supernatants. The data do not conform to a normal distribution. Wilcoxon Test (*: P<0.05, ns: P>0.05).

To explore the relationship between tuft cells and macrophages, we first analyzed their interactions within HAEC. Our findings revealed that they were in proximity to each other (**Figure 5A**). Subsequently, we isolated crypts from C57 mice to culture intestinal organoids, followed by their identification (**Figures 5B–D**). Previous studies have shown that IL-13 promotes the differentiation of tuft cells in intestinal organoids (20, 35). Using IL-13 supplements, we successfully cultured intestinal organoids containing tuft cells (**Figure 5E**), Concurrent CHAT protein expression analysis revealed substantially elevated activation levels subsequent to IL-13 administration(**Figure 5F**). When co-cultured with three different types of macrophages, intestinal organoids containing tuft cells, which were cultured with IL-13 supplements, promoted an increased expression of M2 macrophage markers compared to intestinal organoids without IL-13 supplements (**Figures 5G–I**). Additionally, we observed elevated expression of IL-10, an anti-inflammatory cytokine(**Figure 5J**). These findings suggest that tuft cells not only influence type 2 innate lymphoid cells in the intestine but also affect macrophages.

### 3.6 ACH can promote macrophage polarization towards the M2 phenotype

**Figure 6:**
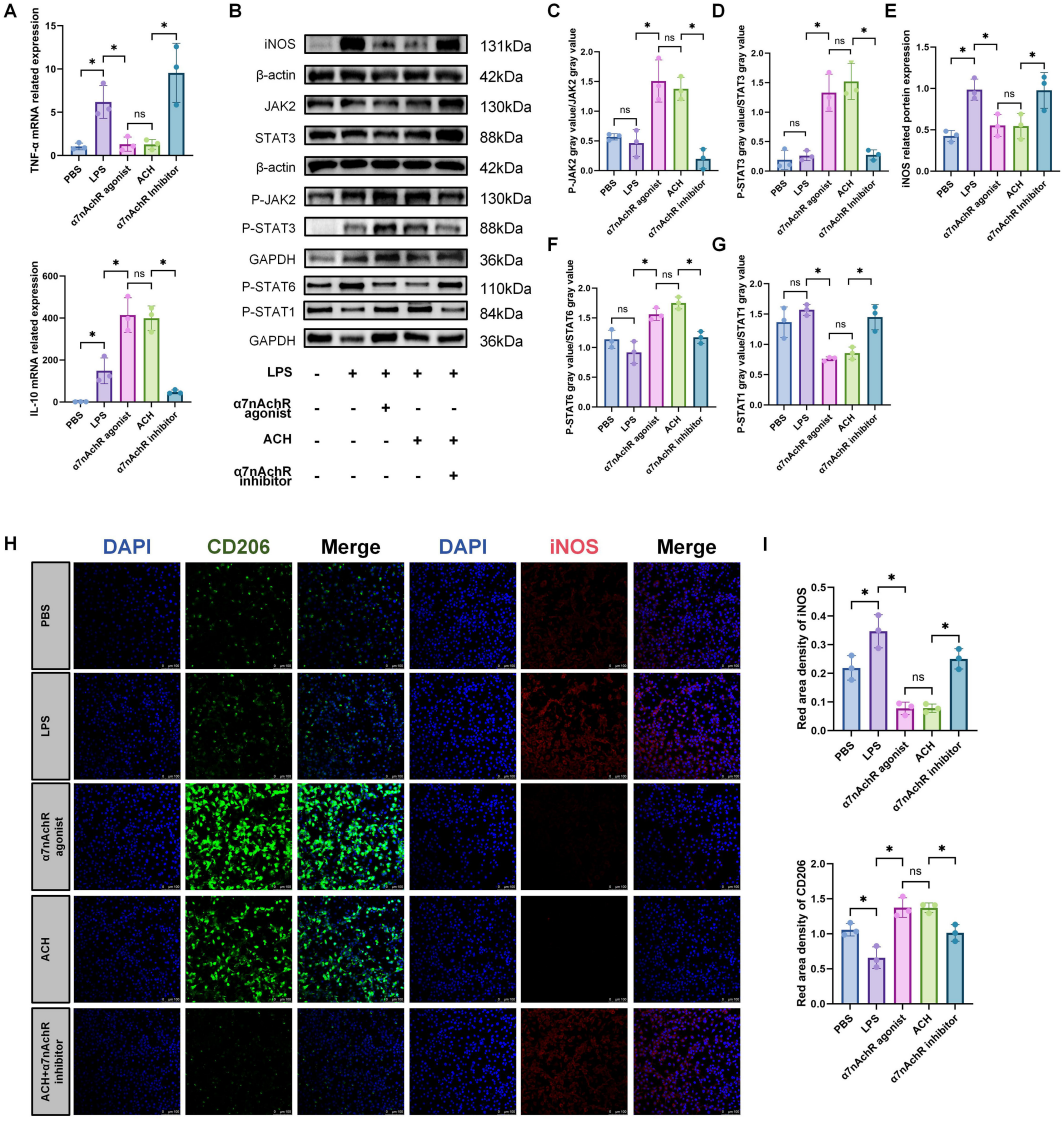
ACH promotes M2 polarization of macrophages to exert an anti-inflammatory effect. **(A)**: In vitro experiments validate the anti-inflammatory effects of ACh on RAW264.7 cells. RAW264.7 cells were pretreated with α7nAchR agonist (10 μM), ACH (1 μM), and α7nAchR inhibitor (10 μM) for 2 hours, followed by stimulation with LPS (0.5 μg/mL) for 22 hours. qRT-PCR was used to validate the expression of inflammation-related genes *TNF-α* and *IL-10*(n=3). The data do not conform to a normal distribution. Mann-Whitney (*: P<0.05, ns: P>0.05). **(B)** Western blotting was performed to assess the activation of inflammation-related proteins iNOS(n=3) and the JAK2-STAT3(n=3) signaling pathway. **(C, D)**.**(E–G)**: Western blotting protein band intensity quantification(n=3). The data do not conform to a normal distribution. Mann-Whitney (*: P<0.05, ns: P>0.05). **(H)**: IF was used to observe the expression levels of M1 macrophage marker (iNOS) and M2 macrophage marker (CD206) in RAW264.7 cells after treatment with LPS, α7nAchR inhibitor, agonist, and ACH(n=3). Red, iNOS; Green, CD206; Blue, DAPI. Scale bars: 100 μm. **(I)**: Statistical analysis of CD206 and iNOS IF optical density in different groups (n=3). All fluorescence intensities across the groups have been normalized to DAPI. The data do not conform to a normal distribution. Mann-Whitney (*: P<0.05, ns: P>0.05).

To explore the anti-inflammatory effects of ACH, we evaluated the expression of genes encoding pro-inflammatory mediators secreted by RAW264.7 cells. The results demonstrated that both ACH and α7nAchR agonists augmented the gene expression of anti-inflammatory factors, including IL-10, and significantly decreased the protein levels of iNOS, along with the expression levels of *TNF-α* genes (**Figure 6A**). Further examination revealed that this anti-inflammatory mechanism was mediated by Activating the JAK2-STAT3 signaling pathway (**Figures 6B–E**). Simultaneously, we observed that STAT1, a polarization marker of M1 macrophages, was activated in the LPS- and α7nAChR inhibitor-treated groups, whereas STAT6, a polarization marker of M2 macrophages, was activated in the α7nAChR agonist- and ACH-treated groups **(** **Figures 6F, G** **)**. Additionally, an increase was observed in the expression levels of markers associated with M2 macrophages in the groups treated with ACH and α7nAchR agonists. Conversely, we observed a decrease in the expression of the markers associated with M1 macrophages (**Figures 6H, I**). These findings indicated that ACH exerts cholinergic anti-inflammatory effects on macrophages.

## 4 Discussion

In this study, inflammation predominantly occurred in the dilated ganglionic segments of HAEC. We observed an abnormally hyperactive cholinergic system in the intestinal epithelium of aganglionic segments. Specifically, through IHC staining, we established that ACHE was abnormally elevated in the intestinal epithelium of the aganglionic segments. Subsequently, we verified that ACH levels were increased in the aganglionic segments of *Ednrb-/-* mice. Finally, by analyzing single-cell RNA data from patients with HSCR, we found that tuft cells were the only intestinal epithelial cells that had the ability to synthesize ACH. Additionally, compared to ganglionic dilated segments, the expression of tuft cell markers was higher in aganglionic segments by IF staining. Considering the established connections between tuft and immune cells in the lamina propria, we investigated whether there were interactions between other immune cells and tuft cells. Moreover, given that the role of macrophages in HAEC has been defined, we believe that a relationship exists between ACH secreted by tuft cells and macrophages. By co-culturing organoids containing tuft cells with three different macrophage lines, we observed an increased expression of M2 macrophage markers, particularly in RAW264.7. This finding is consistent with the phenomenon of elevated M2 macrophage marker expression in the aganglionic intestinal segments. Furthermore, we found that the expression of α7nAchR receptors was significantly increased in the aganglionic intestinal segments of *Ednrb-/-* mice and that these receptors co-localized with macrophages. *In vitro* experiments revealed that ACH and α7nAchR agonists promoted the phenotypic switch of macrophages from M1 to M2. Collectively, these results indicate that in HAEC, ACH in the intestinal epithelium of aganglionic segments is mainly expressed by tuft cells. Tuft cells transmit harmful information from the intestinal lumen to macrophages in the lamina propria by secreting ACH, promoting the M2 polarization of macrophages and thus exerting an anti-inflammatory effect in aganglionic segments.

Pathological examination for ACHE, which indicates the presence of an abnormally hyperactive cholinergic system (36–38), was traditionally established as the gold standard for the diagnosis of HSCR (28). We confirmed this using IHC staining in patients with HSCR, *Ednrb-/-* mice, and BZK-treated rats. Additionally, ELISA experiments performed on *Ednrb-/-* mice revealed a significant increase in ACH expression in the aganglionic segments. Previous studies have demonstrated that RET mutations in HSCR can lead to an increase in CHAT expression in the intestine (39), which partially explains the high expression of ACHE in aganglionic segments. Our findings also revealed a hyperactive cholinergic system in the intestinal epithelial layer. Through analysis of single-cell RNA data from patients with HSCR, we found that tuft cells in the intestinal epithelium were the only cells that could synthesize ACH. Therefore, we believe that ACH of epithelial origin is mainly secreted by tuft cells.

Notably, the BZK rat model has recently emerged as a viable animal model for HSCR (40). Studies have demonstrated elevated ACHE expression in BZK-treated intestinal segments, consistent with the pathological features of HSCR (41). Therefore, the BZK-treated rat model was incorporated into our study as one of the HSCR models. To confirm the success of the BZK rats model, we performed calretinin IHC staining on BZK-treated intestinal segments to check for ganglion cell. Calretinin, a calcium-binding protein, is widely present in the ganglion cells and nerve fibers of the ENS and serves as a neuronal marker (42). In normal intestinal segments, Calretinin exhibits normal staining of ganglion cells and nerve fibers. However, in the aganglionic segment of the bowel affected by HSCR, ganglion cells are absent (43, 44). In this study, the intestinal segments treated with BZK exhibited an absence of ganglion cells, while the distal colon of *Ednrb-/-* mice also displayed aganglionosis. These findings indicated that both models have the same pathological state with HSCR.

Tuft cells are classified as intestinal epithelial secretory cells and are significant members of the intestinal epithelial immune system (26, 45, 46). They are equipped with a series of key components for taste signal transduction, including transient receptor potential melastatin 5 (TRPM5), gustducin, and phospholipase β2 (47–49). These components play central roles in the response of tuft cells to intestinal microbial infections. Therefore, tuft cells represent another major type of intestinal epithelial cell with immune function, alongside goblet cells and Paneth cells (35, 47, 50). Recently, it was found that CHAT, which synthesizes ACH, is expressed in tuft cells of both mice and humans (19). Consequently, tuft cells are regarded as the only epithelial cell type that could synthesize ACH (15, 51). Tuft cells have been detected in patients with HSCR, and compared to ganglionic intestinal segments, the tuft cell marker gene, DCLK1, has been shown to have increased expression in aganglionic segments. This finding aligns with the results of tuft cell IF staining observed in our experiment.

Macrophages play a crucial role in inflammatory progression by polarizing into different types (52, 53). The role of macrophages in HAEC has been well-established; they drive the occurrence of HAEC by promoting polarization towards the M1 phenotype in the dilated segment (54, 55). In our study, we observed an increasing number of M1 macrophages in the ganglionic dilated segment, whereas M2 macrophages were predominant in the aganglionic segment of *Ednrb-/-* mice. Simultaneously, we also observed that the expression of inflammatory factors was predominantly concentrated in the ganglionic dilated segment, with a corresponding decrease in the aganglionic segment. Additionally, we also found that a large number of immune cell infiltrations and crypt abscesses were present in the intestinal epithelium of the ganglionic dilated segment. Therefore, we believe that the inflammation associated with HAEC was mainly concentrated in the dilated ganglionic segment, which is consistent with the findings of previous studies (54, 55). Notably, under normal conditions, IκBα binds to NF-κB and prevents its entry into the nucleus, maintaining NF-κB in an inactive state. When cells receive external stimuli, the IKK complex gets activated and subsequently phosphorylates IκBα at serine residues 32 and 36 (56). Following IκBα phosphorylation, the released NF-κB translocates into the nucleus where it binds to the promoter regions of various inflammation-related genes (57). Therefore, in this study, we performed protein detection of phosphorylated IκBα and revealed that the phosphorylated IκBα expression level was significantly increased in ganglionic dilated intestinal segments.

Studies on tuft cells have primarily focused on their ability to resist helminth infection by promoting the proliferation of ILC2 in the lamina propria mucosa (58). However, in our experiments, IL-13 treatment led to increased tuft cell numbers within the intestinal organoids and upregulated CHAT, co-culturing organoids containing tuft cells with three different macrophage lines resulted in increased expression of M2 macrophage markers, indicating that tuft cells can affect other immune cells. We also observed an increase in IL-10 expression, which was an anti-inflammatory cytokine that could counteract the production of pro-inflammatory factors such as IL-12/23 p40, IL-6 and TNF by macrophages (59), indicating that organoids containing tuft cells could promote M2 macrophage polarization and exert anti - inflammatory effects. IF staining revealed that they were in close proximity to macrophages in *Ednrb-/-* mice. Moreover, the ACH receptor α7nAchR was highly expressed in *Ednrb-/-* mice and co-localized with macrophages. We believe that the tuft cells could promote the polarization of M2 macrophages in the aganglionic segment. Our *in vitro* revealed that ACH increased the expression of M2 macrophage markers, It also promoted the expression of IL-10 gene. ACH activated the JAK2/STAT3 signaling pathway in macrophages, promoting their polarization towards the M2 phenotype, which was consistent with the observations made in the aganglionic segments of *Ednrb-/-* mice.

Concerning the main strengths of this study, we explored the reasons for cholinergic activation in the epithelium of the aganglionic segment and unraveled an acetylcholine-related anti-inflammatory mechanism. However, this study also comprises several limitations. First, we have not explored the acetylcholine synthesis process in tuft cells. Yet, we discovered that IL-13 stimulation boosts acetylcholine synthesis-related CHAT protein expression in organoids. In the future, we will delve deeper into analyzing the acetylcholine synthesis mechanism in tuft cells. Second, though postoperative HAEC exhibits certain incidence after surgery, no such cases occurred in our collected samples. Therefore, our study does not involve postoperative HAEC research. Finally, the organoid experiments in this study were based on normal C57 mice to extrapolate the physiological changes in the intestines of *Ednrb-/-* mice. Given the complexity of the intestinal conditions in *Ednrb-/-* mice, further research is necessary. In the future, we will also focus on developing HSCR-derived intestinal organoids and further elucidating the mechanisms underlying the initiation and progression of HAEC.

In summary, our findings revealed that in HAEC, the hyperactive cholinergic system in the aganglionic segments was associated with increased ACH secretion, which occurred not only in the muscular layer but also in the epithelial layer. Additionally, tuft cells were the only cells in the epithelial layer capable of synthesizing ACH. Therefore, we believe that the elevation of ACH in the epithelial layer of the aganglionic segments is related to the tuft cells. Examination of inflammation in different intestinal segments of HAEC revealed that inflammation in HAEC was predominantly concentrated in the mucosal layer of dilated ganglionic segments. This difference was associated with a reduction in ACH secretion from tuft cells. This reduction potentially hindered the polarization of macrophages into the M2 phenotype. Consequently, it may have contributed to inflammation accumulation and the development of HAEC. Targeting tuft cells to regulate the levels of ACH within the epithelial layer and influence macrophage polarization may become a primary choice for the treatment of HAEC.

## Data Availability

The scRNA-seq data supporting the findings of this study from HSCR intestinal samples have been deposited at Genome Sequence Archive (GSA)(GSA: HRA002993). Any additional information related to the data analysis in this work paper is available from the lead corresponding authors on reasonable request.
